# Associations between medical cannabis and prescription opioid use in chronic pain patients: A preliminary cohort study

**DOI:** 10.1371/journal.pone.0187795

**Published:** 2017-11-16

**Authors:** Jacob M. Vigil, Sarah S. Stith, Ian M. Adams, Anthony P. Reeve

**Affiliations:** 1 University of New Mexico, Department of Psychology, Albuquerque, New Mexico, United States of America; 2 University of New Mexico, Department of Economics, Albuquerque, New Mexico, United States of America; 3 Industrial Rehabilitation Clinics, Albuquerque, New Mexico, United States of America; Pennsylvania State University College of Medicine, UNITED STATES

## Abstract

**Background:**

Current levels and dangers of opioid use in the U.S. warrant the investigation of harm-reducing treatment alternatives.

**Purpose:**

A preliminary, historical, cohort study was used to examine the association between enrollment in the New Mexico Medical Cannabis Program (MCP) and opioid prescription use.

**Methods:**

Thirty-seven habitual opioid using, chronic pain patients (mean age = 54 years; 54% male; 86% chronic back pain) enrolled in the MCP between 4/1/2010 and 10/3/2015 were compared to 29 non-enrolled patients (mean age = 60 years; 69% male; 100% chronic back pain). We used Prescription Monitoring Program opioid records over a 21 month period (first three months prior to enrollment for the MCP patients) to measure cessation (defined as the absence of opioid prescriptions activity during the last three months of observation) and reduction (calculated in average daily intravenous [IV] morphine dosages). MCP patient-reported benefits and side effects of using cannabis one year after enrollment were also collected.

**Results:**

By the end of the 21 month observation period, MCP enrollment was associated with 17.27 higher age- and gender-adjusted odds of ceasing opioid prescriptions (CI 1.89 to 157.36, p = 0.012), 5.12 higher odds of reducing daily prescription opioid dosages (CI 1.56 to 16.88, p = 0.007), and a 47 percentage point reduction in daily opioid dosages relative to a mean change of positive 10.4 percentage points in the comparison group (CI -90.68 to -3.59, p = 0.034). The monthly trend in opioid prescriptions over time was negative among MCP patients (-0.64mg IV morphine, CI -1.10 to -0.18, p = 0.008), but not statistically different from zero in the comparison group (0.18mg IV morphine, CI -0.02 to 0.39, p = 0.081). Survey responses indicated improvements in pain reduction, quality of life, social life, activity levels, and concentration, and few side effects from using cannabis one year after enrollment in the MCP (ps<0.001).

**Conclusions:**

The clinically and statistically significant evidence of an association between MCP enrollment and opioid prescription cessation and reductions and improved quality of life warrants further investigations on cannabis as a potential alternative to prescription opioids for treating chronic pain.

## Introduction

Opioid-related drug overdoses, the current leading cause of preventable deaths in the United State (U.S.), kill roughly 91 Americans every day.[1,2] Conventional pharmaceutical medications for treating opioid addiction, such as methadone and buprenorphine-tapering, can be similarly dangerous due to substantial risks of lethal drug interactions and overdose.[1,3,4] Other medications aimed at reducing the adverse symptoms of opioid withdrawal (e.g., α2-adrenergic agonists, benzodiazepines, antiemetics, non-steroidal anti-inflammatory agents) are not designed to treat patients’ underlying health condition, often chronic pain,[5] leaving patients with limited long-term pharmaceutical treatment options. Whole, natural *Cannabis sativa* and extracts made from the plant may serve as an alternative to opioid-based medications for treating chronic pain.

Cannabis has been investigated as a potential treatment for a wide range of medical conditions from post-traumatic stress disorder[6] to cancer,[7,8] with the most consistent support for the treatment of chronic pain, epilepsy, and spasticity.[9–11] In the U.S., states have enacted Medical Cannabis Programs (MCPs) in part for people with chronic, debilitating pain who cannot be adequately or safely treated with conventional pharmaceutical medications. MCPs are unique, not only because they allow patients to self-manage their cannabis treatment, but because they operate in conflict with U.S. federal law, making it challenging for researchers to utilize conventional research designs to measure their efficacy. To date, no study has used a randomized controlled trial to measure the effects of MCP enrollment, and hence the ability to legally self-manage cannabis therapy, on patient outcomes. State-level analyses, show that the implementation of MCPs is correlated with clinically and statistically significantly lower opioid-related mortality rates and reductions in the number and cost of prescription medications used by Medicare patients,[12–14] suggesting that some patients use cannabis as a substitute for conventional pharmaceutical treatments.[15–17]

New Mexico (NM) is among the U.S. states hardest hit by the current opioid epidemic, although the number of opioid-related overdose deaths appears to have fallen in recent years,[2] perhaps the result of increased enrollment in the NM MCP, which currently includes nearly 50,000 patients. Using informal surveys of patients enrolled in the MCP, we discovered a significant proportion of chronic pain patients reported substituting their opioid prescriptions with cannabis for treating their chronic pain. In this preliminary study, we tested that possibility using a historical, cohort research design in which we measured how opioid prescription patterns compared between habitual prescription opioid-using chronic pain patients who self-selected into the NM MCP and patients that declined the option to enroll in the MCP and did not use cannabis during the same time period, using opioid prescription records as a proxy for opioid consumption. A recent study conducted in Israel found that physician-directed cannabis treatment was associated with improved functionality, reduced pain, and fewer opioid prescriptions in cannabis-naïve, chronic pain patients at a six month follow-up.[18] The current study advances that work by establishing a reliable measurement of pre-MCP enrollment (baseline) opioid prescriptions, comparing MCP enrollees to a medically and demographically similar group of non-enrolled and non-cannabis-using patients, and observing both groups for an average of 21 months.

## Methods

### Study design

This study was approved by the Institutional Review Board at the University of New Mexico; the data was treated confidentially, and the IRB waived the need for patient consent. Using a historical, cohort research design, we measured the effect of enrollment in the NM MCP on opioid prescription patterns in response to patient reports of changes in pain reduction and improved quality of life among enrollees. As in other states, New Mexico only permits medical cannabis use for patients with certain debilitating medical conditions. All the patients in our study had a diagnosis of “severe chronic pain,” annually validated by two independent physicians, including a board-certified specialist (APR).

Although enrollment in the MCP requires physician referrals, once enrolled, patients can self-manage the potency, frequency, and particular type of cannabis product used (e.g., strain of whole dried flower, edible, or extract) in place of, or in conjunction with, conventional pharmaceutical medication treatments. The single-physician rehabilitation clinic (Albuquerque, NM) where the study took place provides all eligible patients with the opportunity to enroll in the MCP, an option which roughly one third of chronic pain patients decide to explore; all of the patients in the current study were invited to enroll in the MCP by the same referring physician between 2010 and 2015. Patients who decided to enroll in the MCP received no direct medical supervision over their cannabis treatment, and were not provided any explicit instructions for altering (e.g., reducing) their opioid prescription usage in line with the clinic’s approach to providing palliative care through patient education and self-management of available treatment options. Although all the patients in the study were receiving treatment for chronic pain at the single-physician (APR) rehabilitation clinic it was common for patients to receive opioid prescriptions from multiple physicians at other clinics (S1 Text).

The MCP patients in the current study were initially recruited to complete an informal survey regarding their experience with the MCP at the time of the medical examination necessary for renewal of their MCP license. One hundred and forty-six patients who enrolled in the MCP between 4/1/2010 and 10/3/2015 completed the surveys. In order to assess opioid prescription patterns among these patients, records from the New Mexico Prescription Monitoring Program were retrieved for the 21 month period spanning from three months pre-enrollment through 18 months post-enrollment. All opioid prescription medications in the Prescription Monitoring Program data were first normalized into milligrams of IV morphine using the GLOBALRPh equivalency calculator,[19] and intravenous morphine (IV, 3:1 oral dosage equivalency) was used to measure patient-level opioid consumption levels.

To create a comparison group, patients from the same rehabilitation clinic that met the following criteria were initially selected: having a diagnosis of one of the three most common chronic back pain conditions; ICD-10 codes: M54.5 (chronic low back pain, multiple sites in spine), M54.2 (cervicalgia, cervical spine/neck pain), or M96.1 (post-laminectomy, failed decompression surgery syndrome); having been offered and declining the invitation to enroll in the MCP between 2010 and 2015; and no current usage of cannabis (verified by random urine analyses conducted approximately every six months during the observation period). This resulted in an initial sample of 53 non-enrolled patients whose prescription records were retrieved over a 21-month period dating from 10/08/2014 through 07/06/2016.

Next, from our initial pool of 146 MPC and 53 non-MCP patients, we included in our sample only patients filling at least two opioid prescriptions during the three months prior to enrollment for the MCP patients and during the first three months of observation for the comparison group, patients with musculoskeletal disorders, and patients with maximum daily dosages of less than 200 mg IV morphine. Eighty-seven MCP patients and fifteen patients in the comparison group did not fill any opioid prescriptions during the first three months and were excluded from the analyses. An additional 18 MCP patients and 9 comparison group patients filled only one opioid prescription in the first three months and were also excluded. One rheumatoid arthritis patient was dropped from the MCP group as having an inflammatory, rather than musculoskeletal disorder. Three other MCP patients received maximum dosages of over 200 mg IV morphine and were dropped as outliers and due to a high possibility of diversion. (S1 Table)

The final sample of habitual opioid users included 37 MCP patients with chronic musculoskeletal and predominantly back pain disorders (mean age = 54 years; 54% male; 86% back pain, 5% knee pain, 3% hip pain, 3% wrist pain, and 3% shoulder pain) and 29 comparison group patients (mean age = 60 years; 69% male; 100% back pain). Due to a recent enrollment date, PMP records were available for only sixteen months for one MCP patient. Four MCP patients had records available for 19 months, seven for 20 months and the remaining 25 for a full 21 months, resulting in an average of 20.6 (± .90) months of observation. All comparison group patients had a full 21 months of Prescription Monitoring Program records retrieved.

### Study outcomes

In our patient-level analysis, *Ceased Opioid Prescriptions* is a dichotomous {0,1} variable with “0” defined as no Prescription Monitoring Program evidence of an opioid prescription filled during the last three months of observation and “1” defined as any opioid prescription filled during that time period. *Reduction in Prescribed Daily Opioid Dosage* measures whether the average prescribed daily dosage of IV morphine was lower in the last three months than in the first three months of observation (pre-enrollment for MCP patients). *Percentage Point Change in Prescribed Daily Opioid Dosage* measures the difference between the average daily dosage in the first and last three months of observation divided by the average daily dosage in the first three months of observation.

In our longitudinal analysis, we converted the prescription-level data from the Prescription Monitoring Program into a patient-month level panel by aggregating dosages and quantities across prescriptions filled each month (for each patient) in order to generate a mean daily dosage per month (mg IV morphine.)

The survey questions (S2 Text) measured existence of side effects, pain levels prior to and following enrollment in the MCP, and effects of cannabis use on quality of life, social life, activity levels, and concentration.

### Statistical analysis

We used a logistic regression model to estimate the effect of MCP participation on cessation of prescribed opioids (absence of opioid prescriptions coded “0”, presence of an opioid prescription coded “1”) and on whether or not patients reduced their average daily prescribed dosage between the first three months (pre-enrollment for MCP patients) and the last three months of observation. A least squares approach was used to analyze the effect of MCP participation on the percentage change in opioid prescriptions. In all cases, we control for age and gender. We report odds ratios for the dichotomous outcomes, and for the percentage change in opioid prescriptions, the coefficient is reported. Reported 95% confidence intervals are based on heteroskedasticity-robust standard errors.

A least squares approach also was used for the longitudinal analysis of the monthly change in the daily dosage (mg IV morphine) associated with MCP participation. We regressed the daily dosage on MCP participation, a month-level trend, and the interaction between MCP participation and a month-level trend, controlling for gender and age. Given the disparity in the average starting opioid prescription dosages, we also analyzed the within-patient differences in opioid prescriptions over time, controlling for time-invariant patient-level characteristics through the use of patient-level dummy variables. Because group participation does not vary over time, we include only MCP patients in the within-patient analysis. Standard errors were clustered at the patient-level to control for heteroskedasticity and arbitrary correlation.

Due to the small sample size, limited covariates, and the lack of variation in many of the survey responses, we used simple univariate hypotheses tests and graphical analyses for these outcomes.

Statistical analyses were conducted using Stata/SE 13.1.

## Results

### Reductions, cessation, and trends in opioid prescription patterns

The descriptive information in Table 1 suggests that MCP patients were more likely either to reduce daily opioid prescription dosages between the beginning and end of the sample period (83.8% versus 44.8%) or to cease filling opioid prescriptions altogether (40.5% versus 3.4%). The percentage point change in daily opioid prescription dosages also differed between the two groups with MCP patients reducing their dosages by 47 percentage points, while the comparison group increased dosages by 10.4 percentage points.

**Table 1:** Effect of MCP enrollment on opioid prescription patterns (Means comparison).

Variable (N = 66)	Comparison (N = 29)	MCP (N = 37)	P Value
Ceased opioid prescriptions {0,1}	3.4% (1)	40.5% (15)	<0.001
Reduced prescribed daily opioid dosage {0,1}	44.8% (13)	83.8% (31)	0.001
Average daily opioid dosage in the 1^st^ 3 months (mg)	16.2 ± 14.8	24.4 ± 23.3	0.103
Average daily opioid dosage in the last 3 months (mg)	12.3 ± 12.4	12.4 ± 20.1	0.974
Change in prescribed daily opioid dosage (mg)	-3.9 ± 13.2	-12.0 ± 23.4	0.101
Percentage point change in prescribed daily opioid dosage	10.4 ± 114.9	-47.0 ± 63.1	0.013
Male	54.1% (20)	69.0% (20)	0.219
Age	59.7 ± 13.8	53.6 ± 9.5	0.036

Notes: For dichotomous variables, the percent and count (in parentheses) are listed; for continuous variables, the mean ± standard deviation. P-values are from chi-squared tests for dichotomous variables and two-sided t-tests for continuous variables. *Ceased opioid prescriptions* means that the patient did not fill any prescriptions for opioid medications during the last three months of observation. *Reduced prescribed daily opioid dosage* is a {0,1} outcome for whether or not a patient reduced their average daily opioid dose (mg) between the first three months of observation and the last three months of observation. *Percentage point change in prescribed daily opioid dosage* compares the average daily opioid dose (mg) between the first three months of observation and the last three months of observation, using the first three months as the baseline.

The odds ratios reported in Panels A and B of Table 2 support the simple means comparison results from Table 1. Not surprisingly, given that only one member of the comparison group ceased their prescriptions of opioids by the end of the sample period, the odds of an MCP patient ceasing opioid prescriptions by the last three months of observation is much larger than that of the comparison group (OR 17.27, CI 1.89 to 157.36, p = 0.012). The odds ratio comparing reduction {0,1} in daily opioid prescription dosages between MCP patients and the comparison group also is statistically and clinically significant at 5.12 (CI 1.56 to 16.88, p = 0.007). The analysis of the percentage point change in daily opioid prescription dosages in Panel C almost matches the results of the means comparisons in Table 1 with MCP patients reducing their daily dosages by 47.13 percentage points relative (p = 0.034) to the comparison group (mean = 10.4% increase), suggesting that the effect of the MCP on opioid prescription patterns did not vary significantly by gender or age in our sample.

**Table 2:** Effect of MCP enrollment on opioid prescription patterns (Regression analysis).

**Panel A: Ceased Opioid Prescriptions {0,1}**		
*Variable*	*OR (95% CI)*, *Baseline Odds = 1*.*00*	*P Value*
MCP	17.27 (1.89 to 157.36)	0.012
**Panel B: Reduced Prescribed Daily Opioid Dosage {0,1}**		
*Variable*	*OR (95% CI)*, *Baseline Odds = 1*.*00*	*P Value*
MCP	5.12 (1.56 to 16.88)	0.007
**Panel C: Percentage Point Change in Prescribed Daily Opioid Dosage**		
*Variable*	*Percentage point change (95% CI)*	*P Value*
MCP	-47.13 (-90.68 to -3.59)	0.034
**Panel D: Patient-Month Level Trend Analysis—MCP versus Comparison Group**		
*Variable*	*Change in mg IV morphine (95% CI)*	*P Value*
MCP	12.47 (2.48 to 22.46)	0.015
Trend (month-level)	0.18 (-0.02 to 0.39)	0.081
MCP*Trend	-0.64 (-1.10 to -0.18)	0.008
**Panel E: Timing of Reduction in Prescribed Daily Opioid Dosage among MCP Participants**		
* *	*Change in mg IV morphine (95% CI)*	*P Value*
Months		
4 to 6	-4.37 (-12.73 to 3.99)	0.296
7 to 9	-7.40 (-15.85 to 1.04)	0.084
10 to 12	-9.67 (-17.42 to -1.93)	0.016
13 to 15	-8.88 (-16.58 to -1.19)	0.025
16 to 18	-10.15 (-17.97 to -2.32)	0.013
19 to 21	-11.43 (-19.32 to -3.53)	0.006

Notes: In Panel A, *Ceased Opioid Prescriptions* means that the patient did not fill any opioid prescriptions in the last three months of observation. In Panel B, *Reduced Prescribed Daily Opioid Dosage* means the patient reduced their average daily dosage of prescribed opioids (Table 2 continued) between the first three months of observation and the last three months of observation. In Panel C, *Percentage Point Change in Opioid Prescriptions* equals the mean prescribed daily dosage during the last three months of observation minus the mean prescribed daily dosage during the first three months of observation divided by the mean prescribed daily dosage in the first three months of observation; the percentage point change associated with MCP enrollment is reported. In Panel D, the linear trend analysis shows the change in milligrams of IV morphine over time associated with MCP enrollment, starting with enrollment for the MCP patients and at the beginning of the fourth month of observation for the comparison group. Panel E shows fixed-effects regressions (at the patient level) of the mean prescribed daily dosage on three month groups starting with the pre-enrollment period and continuing through 18 months post-enrollment. The reported coefficients show the effect on mean prescribed daily opioid dosages of being within a given three month period post-enrollment relative to being in the three month pre-enrollment period. Standard errors are robust to heteroskedasticity in Panels A through C and are robust to heteroskedasticity and intra-patient arbitrary correlation in Panels D and E.

**Fig 1:**
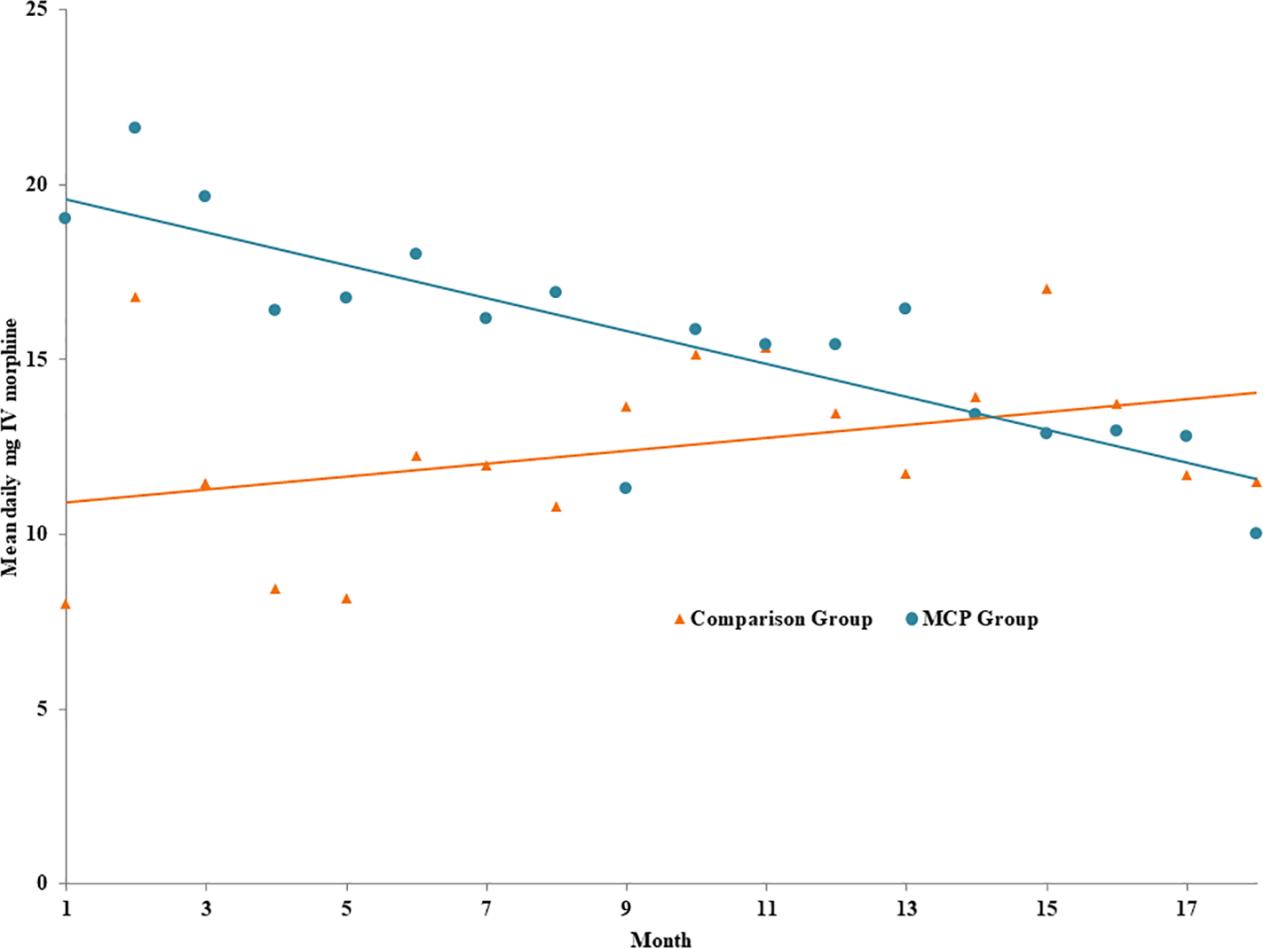
Mean prescribed daily opioid dosage by month. Notes: Month “1” represents the first month post-enrollment for the MCP patients (n = 37) and the fourth month of observation for the comparison group (n = 29). The time trends add a linear representation of the relative change in prescribed daily opioid dosage starting with the time of treatment (enrollment in the MCP).

In order to capture the variation over time in the Prescription Monitoring Program records for each patient, we also created a patient-month level dataset. Fig 1 shows the relationship in the raw data between time (measured in months) and average daily opioid prescription dosage of IV morphine (mg) using a scatter plot. Linear time trends were overlaid for each group. According to Fig 1, the MCP patients appear to have started at higher dosages than the comparison group, but while the MCP patients’ opioid prescription dosages declined over time, the comparison group’s stayed flat or may even have increased slightly. The regression results in Panel D and E of Table 2 further explore this longitudinal relationship. Panel D replicates Fig 1, adjusting for age and gender. MCP patients started at higher prescribed dosages than the comparison group (12.47mg IV morphine higher, CI 2.48 to 22.46, p = 0.015) and although the overall effect of the linear trend is positive (0.18mg IV morphine, CI -0.02 to 0.39, p = 0.08), albeit statistically insignificant, the coefficient on the linear time trend-MCP interaction shows a 0.64mg reduction in the daily opioid prescription dosage of IV morphine per month (CI -1.10 to -0.18, p = 0.008) among MCP patients. Combining the trend coefficients implies that on average, MCP patients were prescribed a lower average daily dosage of IV morphine than the comparison group after about 15.2 months of enrollment ([12.47/(0.64+0.18)] = 15.21 months.)

Panel B showed that MCP patients had higher odds of reducing daily opioid prescription dosages during our observation period. The results in Panel E indicate how the reduction in opioid prescriptions among MCP patients occurred over time by comparing across three month groups, controlling for time-invariant patient characteristics. The coefficient for the first three months post-enrollment is negative, but statistically insignificant; for the fourth through sixth month post-enrollment, the coefficient is larger but still statistically insignificant at conventional levels. For the following three month periods, from month 7 through month 18 post-enrollment, all coefficients are large, negative, and statistically significant. The coefficients also increase in size and show that by the last three month group (months 19 through 21), the average MCP patient was prescribed 11.43 fewer milligrams of IV morphine per day than she or he was in the pre-enrollment period.

We conducted robustness checks adjusting our inclusion criteria to ensure a closer match between our MCP patients with our Comparison patients. Although any restriction reduces the already small sample size, we found that restricting the sample to only back pain patients (8 MCP patients excluded), to only patients between the ages of 30 and 70 (2 MCP patients and 6 Comparison patients excluded), or using a stricter criteria for outliers (mean daily dosage in the first three months of greater than 48mg, which gives a similar mean daily dosage across the MCP and Comparison groups and excludes six MCP patients) does not materially affect the results in terms of magnitude and statistical significance.

In all our regressions, the 95% confidence intervals for our coefficients are large, i.e., while the sign of our coefficients is reliable, we cannot make precise predictions regarding the size of the effect. This could be an artifact of our small sample size or an indication that the magnitude of the effect of MCPs on opioid prescription patterns may vary substantially across individuals even among our relatively homogeneous patients.

### Pain, quality of life, and side effects of using cannabis

**Fig 2:**
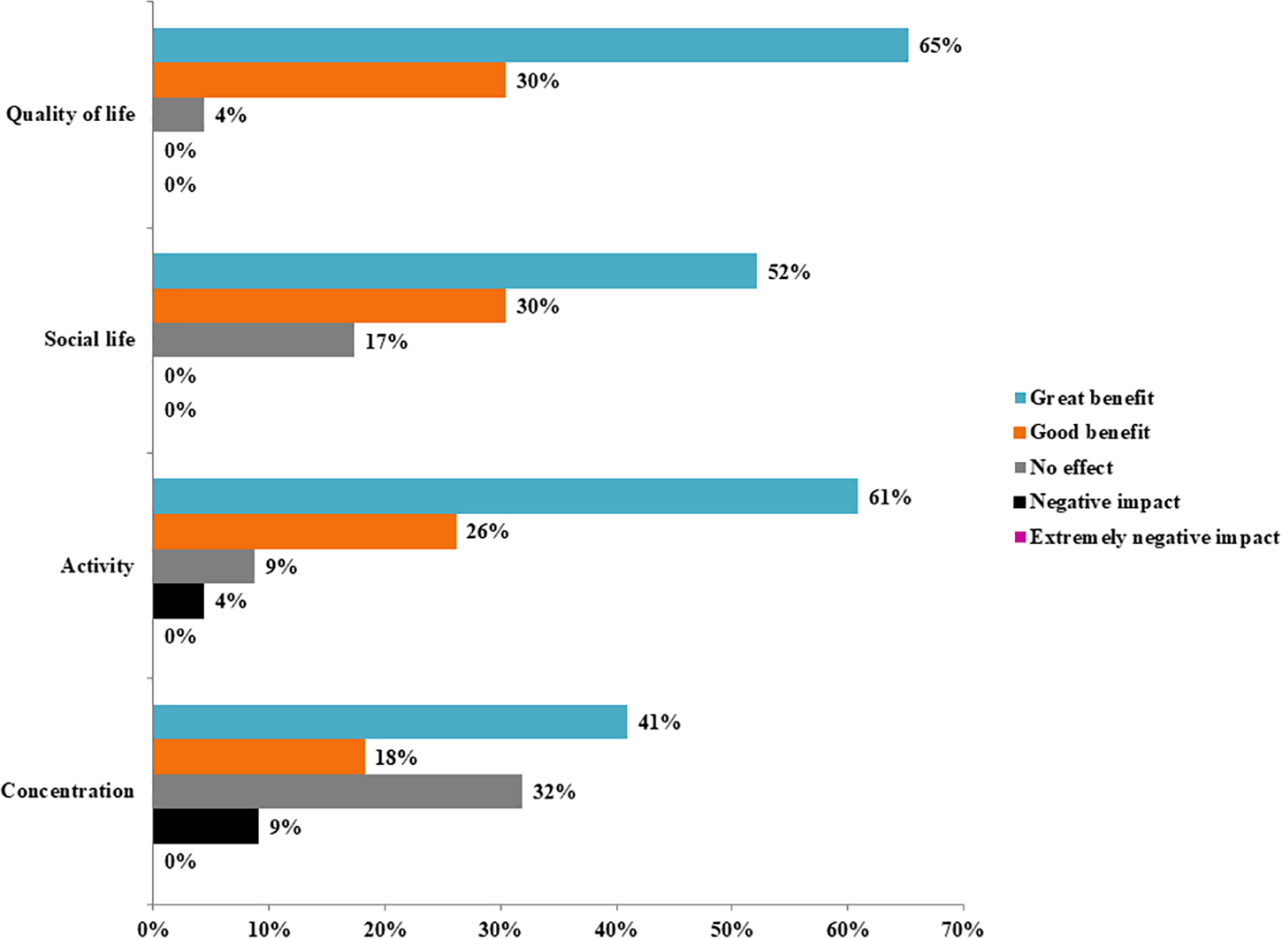
Effects of medical cannabis on quality of life, social interactions, activity levels and concentration. Notes: These survey questions were administered as a follow-up survey to 23 of the 37 MCP patients with frequent use of prescription opioids. No “extremely negative” impacts were reported by any patient, and a “negative impact” was reported by one patient for *Activity* levels and by two patients for *Concentration*.

Table 3 shows that respondents reported pain reduction from self-administered cannabis use (33 of 34 patients) and a statistically significant change in pain levels from pre- to post-enrollment (mean change = -3.4 on a scale of 0 to 10, *p*<0.001). No respondents reported any serious side effects from cannabis use. Fig 2 shows that no patient reported “negative” or “extremely negative” effects on *Quality of Life* (n = 23) or *Social Life* (n = 23), all but three patients reported a “good” or “great benefit” in their *Activity Level* (n = 23), and the majority reported an improvement in *Concentration* (n = 22) relative to prior to enrollment in the MCP.

**Table 3:** Survey responses at one year Post-MCP enrollment.

Variable	N	Mean ± SD	Min	Max	Null Hypothesis	P Value
Pain reduction from Cannabis usage (Yes = 1/No = 0)	34	0.97 ± 0.17	0	1	Pain reduction = 0	<0.001
Pain prior to Cannabis Program (0 to 10)	34	8.6 ± 1.4	4	10	Pain prior = 0	<0.001
Pain after Cannabis Program (0 to 10)	34	5.3 ± 1.7	2	10	Pain post = 0	<0.001
Change in pain (pain post—pain prior)	34	-3.4 ± 2.1	-7	3	Change< = 0	<0.001
Side effects from Cannabis usage (Yes = 1/No = 0)	34	0	0	0	Side effects = 1	.

Notes: Yes/No responses were coded as yes = 1, no = 0. Pain scale ranges from 0 (pain free) to 10 (worst pain).One-sided t-tests were performed for ranked variables, and chi-squared tests were used for yes/no responses.

## Discussion

The current preliminary findings build upon recent studies suggesting that medical cannabis laws result in clinically and statistically significant (up to 33%) reductions in opioid-related causes of death [12,13] and opioid usage.[14,18] We showed that MCP enrollment, and thus having the legal ability to self-manage cannabis therapy, reduced opioid prescriptions filled by a small sample of habitual opioid-using, non-cancer chronic pain patients. Our observational study circumvented some of the limitations to external validity found in the existing literature, e.g., a lack of comparison group, accurate baseline dosage, and/or potencies much lower than those found in products typically used by NM MCP patients.[20,21] Consistent with previous work suggesting that patients may substitute cannabis for prescription opioids,[14–17] over 80% of the MCP enrollees in our sample reduced their daily opioid prescription dosages, and over 40% ceased filling opioid prescriptions altogether by 1.5 years after enrollment. In addition, MCP patients consistently reported reduced pain and improved quality of life, social interactions, and activity levels as a result of their participation in the MCP.

The relative safety and efficacy of cannabis in comparison to that of opioid prescriptions filled by the patients in our sample is beyond the scope of this study. However, one potential interpretation of our results is that cannabis enables opioid-using patients to engage in their own method of harm reduction. Several meta-analyses have shown that opioids provide only modest immediate pain relief in people with lower back pain,[22–24] and no documented evidence exists of long-term benefits for treating chronic pain.[25,26] Moreover, due to an intense public safety campaign against the dangers of opioid prescriptions in the U.S., patients might be increasingly aware of the associated risks including lethal overdose, abuse, behavioral accidents, myocardial infarction, and other side effects (e.g., gastrointestinal and sexual dysfunctions).[25,27] In contrast, some recent studies have suggested that smoked *Cannabis sativa* flower may be relatively safer for long-term use in adults,[28,29] generally well-tolerated by patients,[7,30] and is not associated with an increased risk of mortality in people with or without comorbid opioid, alcohol, or cocaine use disorders.[31–33] While numerous short-term adverse events can result from cannabis use (e.g., dizziness, confusion, disorientation, loss of balance, somnolence, hallucinations), the majority of patient-reported side effects appear to be non-serious.[11,34] It remains unstudied how cannabis-based products (e.g., edibles, concentrates) and the extraction methods and solvents used to make such products affect patient health.

Our study does have limitations, including the small sample size (underpowered statistical analyses) and observational nature (unmeasured confounding) of the study, likely affecting the extent of its generalizability to other patient groups. Under federal law, we could not randomly assign patients to the MCP; rather patients self-selected into the program and then into renewal, introducing possible selection bias if our comparison group differs fundamentally from our MCP patients in ways that affect opioid consumption over our sample period. The comparison group patients had previously declined referral for medical cannabis, though the reasons are unknown but could include previous ineffective use of cannabinoids, as well as social or financial constraints. Publicly available data from the MCP indicates that 88% of patients who enrolled in the MCP in 2014 reenrolled in 2015, and reasons to not reenroll could include not only a failure to experience therapeutic benefits, but also such significant benefits that cannabis is no longer necessary or external factors such as social pressures or employment concerns. Yet another possible source of selection bias is the statistically significant difference in baseline opioid prescriptions between the two groups, with the MCP patients starting off with higher average daily opioid dosages than the non-MCP patients during the first three months of observation. It may be that the average patient must be prescribed a relatively high average daily dose before she or he is willing to consider enrolling in an MCP. Clearly we cannot rule out selection bias in our between-patient analyses. However, the within-patient (fixed-effects) analyses support that, even after controlling for time-invariant patient characteristics, at least those patients inclined to join an MCP show a statistically and clinically significant reduction in opioid consumption, as measured by their prescribed average daily dose.

Another limitation was that the inclusion of only patients who renewed their MCP licenses likely oversampled patients with an incentive to convince their medical referee that cannabis had been an effective treatment. Although reported attrition in the MCP was low, we were unable to measure it directly. We also were only able to measure MCP enrollment and opioid prescriptions filled, not actual cannabis and opioid consumption, and diversion or hoarding may have existed for both medications. Urine drug screening was also not used to verify cannabis or opioid use in the MCP group due to the nature of the study design (i.e., patient-generated claims that resulted in the construction of a historical cohort study). Without a longer pre-enrollment observation period, we were not able to establish pre-existing trends in opioid usage, and it is possible that some MCP patients were already reducing their consumption in conjunction with seeking alternative treatments or as a result of regression towards the mean, although mean reversion would not explain the group differences in cessation rates. Our pain assessment method was also limited by its non-standardized, informal, and retrospective nature, and was only completed by the MCP patients. Finally, the generalizability of our study is likely limited in part due to the small number of patients in our sample; a single state MCP and clinic; and demographic characteristics specific to our sample. Caution should therefore be taken in extending the results of our study to populations thought to be at greater risk of negative side effects.[35] Future, larger investigations should attempt to target causation and variant patterns (e.g., rates of change) of medication usage, while controlling of relevant individual-level factors (e.g., life-histories, current social environments, setting of substance usage), and incorporate more comprehensive bio- and psychometric outcomes/effects assessments through the implementation of creative research designs that can operate around the current federal barriers for conducting medical cannabis research in the U.S.[20]

Despite the current study’s limitations, the results from this preliminary study showed a strong correlation between enrollment in an MCP and cessation or reduction of opioid use. From a harm reduction standpoint, our results highlight the necessity of more extensive research into the possible use of cannabis as a substitute for opioid painkillers, especially in the form of placebo-based, randomized controlled trials and larger sample pragmatic studies.[36] The economic impact of cannabis treatment may also be considered given the current burden of opioid prescriptions on healthcare systems, which have been forced to implement costly modifications to general patient care practices, including prescription monitoring programs, drug screening, more frequent doctor-patient interactions, treatment of drug abuse and dependence, and legal products and services associated with limiting opioid-related liability.[37] In summary, if cannabis can serve as an alternative to prescription opioids for at least some patients, legislators and the medical community may want to consider medical cannabis programs as a potential tool for combating the current opioid epidemic.[14,38]

## Supporting information

S1 Text Study context and setting. (DOCX)

S2 Text Survey questions. (DOCX)

S1 Table Sample selection. MCP patients include all patients willing to complete the survey; non-MCP patients were selected based on a diagnosis of back pain, refusal of an MCP referral, and no evidence of cannabis usage in urine drug screens.
(DOCX)

## References

[1] CDC. Wide-ranging online data for epidemiologic research (WONDER) Atlanta, GA: CDC, National Center for Health Statistics; 2016 Available at http://wonder.cdc.gov.

[2] Rudd RA, Seth P, David F., Scholl L. Increases in drug and opioid-involved overdose deaths—United States, 2010–2015. MMWR Morb Mortal Wkly Rep 2016;65:1445–1452. doi: 10.15585/mmwr.mm655051e1 28033313 10.15585/mmwr.mm655051e1

[3] Crane EH. Emergency department visits involving buprenorphine. The CBHSQ Report: January 29, 2013. Center for Behavioral Health Statistics and Quality, Substance Abuse and Mental Health Services Administration, Rockville, MD. 27606401

[4] Garcia-Portilla MP, Bobes-Bascaran MT, Bascaran MT, Saiz PA, & Bobes J. Long term outcomes of pharmacological treatments for opioid dependence: does methadone still lead the pack? British Journal of Clinical Pharmacology 2012;77:272–284. 10.1111/bcp.12031 PMC4014027 23145768

[5] Schuckit MA. Treatment of opioid-use disorders. The New England Journal of Medicine, 2016;375:357–368. doi: 10.1056/NEJMra1604339 27464203 10.1056/NEJMra1604339

[6] Bonn-Miller MO, Vujanovic AA, & Drescher KD. Cannabis use among military veterans after residential treatment for posttraumatic stress disorder. Psychology of Addictive Behaviors 2011;25:485–491. doi: 10.1037/a0021945 21261407 10.1037/a0021945

[7] Abrams DI. Integrating cannabis into clinical care. Current Oncology 2016: 23(s2); s8–s14. 27022315 10.3747/co.23.3099 PMC4791148

[8] Wilkie G, Sakr B, Rizack T. Medical marijuana use in oncology. A Review. *JAMA Oncol*. 2016;2(5):670–675. doi: 10.1001/jamaoncol.2016.0155 10.1001/jamaoncol.2016.0155 26986677

[9] Devinsky O, Marsh E, Friedman D, et al Cannabidiol in patients with treatment-resistant epilepsy: an open-label interventional trial. Lancet Neurol. 2016; 15:270–278. doi: 10.1016/S1474-4422(15)00379-8 26724101 10.1016/S1474-4422(15)00379-8

[10] Friedman D, Devinsky O. Cannabinoids in the treatment of epilepsy. N Engl J Med 2015;373:1048–58. doi: 10.1056/NEJMra1407304 26352816 10.1056/NEJMra1407304

[11] Whiting PF, Wolff RF, Deshpande S, et al Cannabinoids for medical use: A systematic review and meta-analysis. JAMA 2016;3913(24):2456–2473. 10.1001/jama.2015.6358 26103030

[12] Bachhuber MA, Saloner B, Cunnignham CO, et al Medical cannabis laws and opioid analgesic overdose mortality in the United States, 1999–2010. JAMA Intern Med. 2014;174(10): 1668–1673. doi: 10.1001/jamainternmed.2014.4005 25154332 10.1001/jamainternmed.2014.4005 PMC4392651

[13] Kim JH, Santaella-Tenorio J, Mauro C, et al State medical marijuana laws and the prevalence of opioids detected among fatally injured drivers. American Journal of Public Health 2016;106(11):2032–2037. doi: 10.2105/AJPH.2016.303426 27631755 10.2105/AJPH.2016.303426 PMC5055785

[14] Bradford AC, Bradford WD. Medical marijuana laws reduce prescription medication use in Medicare Part D. Health Affairs. 2016 7 1;35(7):1230–6. doi: 10.1377/hlthaff.2015.1661 27385238 10.1377/hlthaff.2015.1661

[15] Reiman A. Cannabis as a substitute for alcohol and other drugs. Harm Reduct J. 2009 12 3;6:35 doi: 10.1186/1477-7517-6-35 19958538 10.1186/1477-7517-6-35 PMC2795734

[16] Lucas P., Walsh Z., Crosby K., Callaway R., Belle-Isle L., Kay R., Capler R., and Holtzman S. Substituting cannabis for prescription drugs, alcohol and other substances among medical cannabis patients: The impact of contextual factors. Drug Alcohol Rev. 2016, 35: 326–333. doi: 10.1111/dar.12323 26364922 10.1111/dar.12323

[17] Lucas P., & Walsh Z. Medical cannabis access, use, and substitution for prescription opioids and other substances: A survey of authorized medical cannabis patients. International J Drug Policy 2017; 42, 30–5. 10.1016/j.drugpo.2017.01.011 28189912

[18] Haroutounian S, Ratz Y, Ginosar Y, Furmanov K, Saifi F, Meidan R, Davidson E. The effect of medicinal cannabis on pain and quality-of-life outcomes in chronic pain: A prospective open-label study. Clin J Pain 2016;32(12):1036–1043. doi: 10.1097/AJP.0000000000000364 26889611 10.1097/AJP.0000000000000364

[19] Globalrph.com opioid narcotic analgesic converter. GlobalRPh.com 2016. (Accessed January 1, 2017, at http://www.globalrph.com/narcotic.cgi/).

[20] Stith SS, & Vigil JMV. Federal barriers to *Cannabis* research. Science 2016; 352 (6290), 1182. 10.1126/science.aaf7450 27257247

[21] Scientificbasesolutions.com. Average potency level of Cannabis sativa flower tested in 2016. (Accessed April 8, 2017, at http://www.scientificbasesolutions.com/community-based-projects.html)

[22] Deyo RA, Von Korff M, Duhrkoop D. Opioids for low back pain. BMJ. 2015;350:g6380 doi: 10.1136/bmj.g6380 25561513 10.1136/bmj.g6380 PMC6882374

[23] Martell BA, O’Connor PG, Kerns RD et al Opioid treatment for chronic back pain: Prevalence, efficacy, and association with addiction. Ann Intern Med. 2007;146:116–127. 17227935 10.7326/0003-4819-146-2-200701160-00006

[24] Shaheed CA, Maher CG, Williams KA, Day R, & McLachlan AJ. Efficacy, tolerability, and dose-dependent effects of opioid analgesics for low back pain. A systematic review and meta-analysis. JAMA Intern Med. 2016;176(7):958–968. doi: 10.1001/jamainternmed.2016.1251 27213267 10.1001/jamainternmed.2016.1251

[25] Chou R, Turner JA, Devine EB, et al The effectiveness and risks of long-term opioid therapy for chronic pain: a systematic review for a National Institutes of Health Pathways to Prevention Workshop. Ann Intern Med. 2015;162(4):276–286. doi: 10.7326/M14-2559 25581257 10.7326/M14-2559

[26] Trescot A, Glaser SE, Hansen H, Benyamin R, Patel S, & Manchikanti L. Effectiveness of opioids in the treatment of chronic non-cancer pain. Pain Physician 2008;11:S181–S200. 18443639

[27] Chey WD, Webster L, Sostek M, Lappalainen J, Barker PN, & Tack J. Naloxegol for opioid-induced constipation in patients with non-cancer pain. New Engl J Med. 2014; 370:25. 10.1056/NEJMoa1310246 24896818

[28] Hill KP, Weiss RD. Minimal physical health risk associated with long-term cannabis use—but buyer beware. *JAMA*. 2016;315(21):2338–2339. doi: 10.1001/jama.2016.5181 27272585 10.1001/jama.2016.5181

[29] Tashkin DP. Effects of marijuana smoking on the lung. Annals of the American Thoracic Society, Vol. 10, No. 3 (2013), pp. 239–247. doi: 10.1513/AnnalsATS.201212-127FR 23802821 10.1513/AnnalsATS.201212-127FR

[30] Ware MA, Wang T, Shapiro S, Collet J, & COMPASS study team. Cannabis for the management of pain: Assessment of safety study (COMPASS). J Pain 2015;16(12):1233–1242. doi: 10.1016/j.jpain.2015.07.014 26385201 10.1016/j.jpain.2015.07.014

[31] Fuster D, Sanvisens A, Bolao F, et al Cannabis as a secondary drug is not associated with a greater risk of death in patients with opiate, cocaine, or alcohol dependence. J Addiction Medicine. Epub 2016 10 6. 10.1097/ADM.0000000000000266 27753720

[32] Ashton CH. Pharmacology and effects of cannabis: a brief review. Br J Psychiatry 2001;178 (2):101–6. 11157422 10.1192/bjp.178.2.101

[33] Calabria B, Degenhardt L, Hall W, Lynskey M. Does cannabis use increase the risk of death? Systematic review of epidemiological evidence on adverse effects of cannabis use. Drug Alcohol Rev. 2010; 29(3): 318–30. doi: 10.1111/j.1465-3362.2009.00149.x 20565525 10.1111/j.1465-3362.2009.00149.x

[34] Wang T, Collet JP, Shapiro S, Ware MA (6 2008). Adverse effects of medical cannabinoids: a systematic review. *CMAJ* (Review) 178 (13): 1669–78. 10.1503/cmaj.071178 PMC2413308 18559804

[35] National Academies of Sciences, Engineering, and Medicine. 2017 *The health effects of cannabis and cannabinoids*: *The current state of evidence and recommendations for research* Washington, DC: The National Academies Press doi: 10.17226/24625 28182367

[36] Rubin R. Medical marijuana is legal in most states, but physicians have little evidence to guide them. JAMA 2017 4 5 doi: 10.1001/jama.2017.0813 10.1001/jama.2017.0813 28384774

[37] Volkow ND, & McLellan T. Opioid abuse in chronic pain—Misconceptions and mitigation strategies. The New England Journal of Medicine 2016;374(13):1253–1263. doi: 10.1056/NEJMra1507771 27028915 10.1056/NEJMra1507771

[38] Hurd YL. Cannabidiol: Swinging the marijuana pendulum from 'weed' to medication to treat the opioid epidemic. Trends in Neurosciences; pii: S0166-2236(17)30001-2. doi: 10.1016/j.tins.2016.12.006 10.1016/j.tins.2016.12.006 28162799

