# Supplementary material for: Associations between medical cannabis and prescription opioid use in chronic pain patients: A preliminary cohort study
Source: PLoS One. 2017 Nov 16;12(11):e0187795. doi: 10.1371/journal.pone.0187795 (PMC5690609; doi:10.1371/journal.pone.0187795)
Supplement: S1 Text — (DOCX) [file pone.0187795.s001.docx]

**S1. Study context and setting**

The New Mexico Medical Cannabis Program (NMMCP) was created in 2007 under the Lynn and Erin Compassionate Use Act. The purpose of this Act was to “allow the beneficial use of medical cannabis in a regulated system for alleviating symptoms caused by debilitating medical conditions and their medical treatments.” The New Mexico Department of Health administers the MCP “in accordance with the Act while at the same time ensuring proper enforcement of any criminal laws for behavior that has been deemed illicit by the state." The act currently allows for 20 qualifying conditions, several of which involve chronic pain as a primary symptom (e.g., cancer and inflammatory auto-immune-mediated arthritis), as well as two debilitating pain conditions described as “severe chronic pain” and “painful peripheral neuropathy.” Qualifying for medical cannabis to treat chronic pain required diagnoses from two independent NM providers (one being a pain specialist) during our sample period.

The number of patients enrolled in the NMMCP has nearly doubled every year since its inception. This has resulted in a new class of drug, natural cannabis-based products. These have been rapidly introduced into the population without supervision by medical personnel.  The original intent of the study was to evaluate the anecdotal reports of patients discontinuing their opioid medications while on cannabis.  In so doing, the currently study was primarily designed to examine whether those anecdotal reports were, in fact, true and occurring over a broader spectrum of patients rather than simply in a few incidental cases.

Once enrolled in the NMMCP, patients have access to all products sold in state-authorized medical cannabis dispensaries, as well as the ability to personally grow *Cannabis sativa* and manufacture cannabis-based products. No restrictions exist on the types of products sold by dispensaries nor on the ratio of THC to CBD, but Tetrahydrocannabinol (THC) concentrations cannot exceed 70%.
