# Supplementary material for: Associations between medical cannabis and prescription opioid use in chronic pain patients: A preliminary cohort study
Source: PLoS One. 2017 Nov 16;12(11):e0187795. doi: 10.1371/journal.pone.0187795 (PMC5690609; doi:10.1371/journal.pone.0187795)
Supplement: S2 Text — (DOCX) [file pone.0187795.s002.docx]

**S2. Survey questions**

**A. Survey Title: Recertification Survey of Medical Marijuana Use**

1. Have you suffered any side effects from use of Medical Cannabis? (Yes/No)
2. In general has Cannabis reduced your pain levels? (Yes/No)
3. In general what was your pain scale prior to the Cannabis Program? (On a scale of one to ten, with zero measured as pain free and ten being the worst pain)
4. In general what was your pain scale after starting the Medical Cannabis Program (On a scale of one to ten, with zero measured as pain free and ten being the worst pain)

**B. Survey Title: Follow-up questionnaire**

Possible answers: Great benefit, good benefit, no effect, negative impact, extremely negative impact

1. How has cannabis affected your quality of life?
2. How has cannabis affected the ability to enjoy time spent with family and friends?
3. How has cannabis affected your overall activity levels?
4. How has cannabis affected your ability to concentrate?
