# Supplementary material for: Associations between medical cannabis and prescription opioid use in chronic pain patients: A preliminary cohort study
Source: PLoS One. 2017 Nov 16;12(11):e0187795. doi: 10.1371/journal.pone.0187795 (PMC5690609; doi:10.1371/journal.pone.0187795)
Supplement: S1 Table — MCP patients include all patients willing to complete the survey; non-MCP patients were selected based on a diagnosis of back pain, refusal of an MCP referral, and no evidence of cannabis usage in urine drug screens. (DOCX) [file pone.0187795.s003.docx]

**S3. Table: Sample selection**

|  | **Comparison Group** | **MCP Group** |
| --- | --- | --- |
| Total Prescriptions in PMP | 1446 | 2953 |
| Opioid | 891 | 1452 |
| Non-opioid | 555 | 1202 |
| Total patients in initial sample* | 53 | 146 |
| -no opioid prescriptions in first 3 months | 15 | 87 |
| -only one opioid prescription in the first 3 months | 9 | 18 |
| -maximum daily dose > 200 mg IV morphine | 0 | 3 |
| -rheumatoid arthritis | 0 | 1 |
| Total patients in final sample | 29 | 37 |

Note. MCP patients include all patients willing to complete the survey; non-MCP patients were selected based on a diagnosis of back pain, refusal of an MCP referral, and no evidence of cannabis usage in urine drug screens.
